# Metabolomics variable selection and classification in the presence of observations below the detection limit using an extension of ERp

**DOI:** 10.1186/s12859-017-1480-8

**Published:** 2017-02-02

**Authors:** Mari van Reenen, Johan A. Westerhuis, Carolus J. Reinecke, J Hendrik Venter

**Affiliations:** 1 Biosystems Data Analysis, Swammerdam Institute for Life Sciences, University of Amsterdam, Science Park 904, 1098 XH Amsterdam, The Netherlands; 2 Centre for Business Mathematics and Informatics, Faculty of Natural Sciences, North-West University (Potchefstroom Campus), Private Bag X6001, Potchefstroom, South Africa; 3 Centre for Human Metabolomics, Faculty of Natural Sciences, North-West University (Potchefstroom Campus), Private Bag X6001, Potchefstroom, South Africa

**Keywords:** Detection limit, Probability mass at zero, Variable selection, Classification, Metabolomics

## Abstract

**Background:**

ERp is a variable selection and classification method for metabolomics data. ERp uses minimized classification error rates, based on data from a control and experimental group, to test the null hypothesis of no difference between the distributions of variables over the two groups. If the associated p-values are significant they indicate discriminatory variables (i.e. informative metabolites). The p-values are calculated assuming a common continuous strictly increasing cumulative distribution under the null hypothesis. This assumption is violated when zero-valued observations can occur with positive probability, a characteristic of GC-MS metabolomics data, disqualifying ERp in this context. This paper extends ERp to address two sources of zero-valued observations: (i) zeros reflecting the complete absence of a metabolite from a sample (true zeros); and (ii) zeros reflecting a measurement below the detection limit. This is achieved by allowing the null cumulative distribution function to take the form of a mixture between a jump at zero and a continuous strictly increasing function. The extended ERp approach is referred to as XERp.

**Results:**

XERp is no longer non-parametric, but its null distributions depend only on one parameter, the true proportion of zeros. Under the null hypothesis this parameter can be estimated by the proportion of zeros in the available data. XERp is shown to perform well with regard to bias and power. To demonstrate the utility of XERp, it is applied to GC-MS data from a metabolomics study on tuberculosis meningitis in infants and children. We find that XERp is able to provide an informative shortlist of discriminatory variables, while attaining satisfactory classification accuracy for new subjects in a leave-one-out cross-validation context.

**Conclusion:**

XERp takes into account the distributional structure of data with a probability mass at zero without requiring any knowledge of the detection limit of the metabolomics platform. XERp is able to identify variables that discriminate between two groups by simultaneously extracting information from the difference in the proportion of zeros and shifts in the distributions of the non-zero observations. XERp uses simple rules to classify new subjects and a weight pair to adjust for unequal sample sizes or sensitivity and specificity requirements.

**Electronic supplementary material:**

The online version of this article (doi:10.1186/s12859-017-1480-8) contains supplementary material, which is available to authorized users.

## Background

Feature selection and classification in metabolomics can be problematic due to the large number of missing values often present in the data. Specifically, metabolomics data generated through gas chromatography-mass spectrometry (GC-MS), are known to contain many missing values [1]. To complicate matters further, values can be missing at random or not, depending on the source of the missing values. Missing values can result from technical limitations, for example peak misalignment, deconvolution errors resulting from the indistinct shape of a peak, the detection limit of the platform or any combination of these sources. A missing value can also have a biological origin, i.e. a metabolite which is a marker for some disease can be truly absent from a healthy sample. Datasets containing missing values can become cumbersome when performing statistical analysis. Kang [2] lists lack of power and estimation bias as some of the concerns. As a result, many different imputation strategies have been tried and tested, from very basic strategies like replacing missing values by some fraction of the minimum observed value, to more advanced techniques that aim to impute values based on the remaining data. Imputation is however not always ideal or straightforward. The more successful techniques, such as k-nearest-neighbour and random forest [1, 3], require larger sample sizes, a known limitation of metabolomics studies. Other approaches, even the most elementary ones where missing values are replaced by a fixed number, require the tuning or estimation of parameters. It is also ill-advised to make use of a one-size-fits-all approach to dealing with missing values, especially given the different sources of missing values. Armitage et al [3] propose a combination of zero value imputation when the metabolite is assumed absent from a sample for biological reasons and k-nearest-neighbour when missing values are believed to be the result of the technical limitations of the platform. Specifically, applying ERp to data with missing values imputed by random numbers can have some unwanted effects without any real gains, as we show in a comparative study reported in Additional file 1: Section S7.

The research into missing value replacement is vast and we do not go into further detail here, instead we propose a somewhat different approach. Ensuring that the data contains as few as possible missing values due to technical errors must be the first line of defence. Again, we do not go into detail here since software packages are continually being improved and developed to reduce the number of false positives and negatives during peak identification and quantification [4, 5]. The remaining missing values, predominantly resulting from the detection limit of the platform and biological sources, can then reasonably be replaced by zeros. Since a large proportion of zero values still poses a challenge for hypothesis testing, we devise a new test statistic which can accommodate zeros. We first discuss why a new test statistic is needed.

Traditional statistical tests make distributional assumptions or are sensitive to skewed distributions such as those resulting from data with a pronounced frequency of zero values. To control the proportion of zero values a “zero filter” can be applied and entails the removal of variables containing too many zeros from the data [6]. The minimum proportion of zero values required to remove a variable is rather arbitrary, but the common consensus is that the proportion should be high (e.g. at least 50%). This proportion is now a tuning parameter which forms part of any resulting model and this is not ideal. Also, we cannot guarantee that important metabolites will not be discarded even if the group structure is taken into account. Alternatively, an equal number of zero observations can be removed from each group, e.g. the Chop-Lump approach [7] which proved powerful when combined with the Wilcoxon or t-test. However, this approach will further reduce already small group sizes for which metabolomics research is known. More complex approaches have also been proposed and we group them into three categories: (i) one-part tests that account for the mixture distributions of data with a positive probability of zero values [8, 9]; (ii) two-part tests that compare the proportions of zeros and the non-zero values separately [10]; and (iii) inverted survival analysis methods [11]. However, these approaches have their limitations. One- and two-part tests are known to explain the presence of zero values either due to technical (e.g. below detection limit) or biological (e.g. metabolite not present) reasons, but not necessarily both [12]. One- and two-part tests have proved valuable in the variable selection context, but do not have the ability to classify new subjects and constructing classification models as a second phase has been criticized [13, 14]. Furthermore, two-part tests still rely on independently derived and equal weighted test statistics for the zero and non-zero data and, as a result, may lack power [15]. Methods derived from survival analysis have also proven valuable, but require knowledge of the actual detection limit [11].

Our proposed new test statistic is derived from ERp, a recently introduced approach for variable selection and classification with application to metabolomics data [14]. In its current form, ERp makes use of p-values associated with minimized classification error rates to identify variables that can discriminate between a control and experimental group. These p-values are calculated based on the assumption that the cumulative distribution function (CDF) for the two groups, common under the null hypothesis, starts at zero at the lower limit of its range of values and continuously increases to one, at the upper limit of its range. This assumption does not allow for jumps in the CDF and in particular, does not cater for a positive probability at zero. In this paper we introduce XERp, an extension of ERp, which takes the presence of two sources of zeros into account: (i) zeros representing instances where the metabolite is truly absent from the biological sample (e.g. metabolites depleted or expressed by a disease); and (ii) zeros representing observations below the detection limit of the metabolomics platform used. Low level metabolites of some diseases, which do not have a monogenetic origin, are mostly not reflected as major metabolic perturbations characterized by dominant metabolic biomarkers. These diseases, of which tuberculosis meningitis is an example and the data used in this study, are of particular interest due to their importance in community health. ERp in its current form can already accommodate many of the characteristics of metabolomics data such as small sample sizes, unequal group sizes and data without dominant biomarkers, making XERp an important and logical extension through its accommodation of zero values.

In the Methods section, we show how the null distributions used in ERp can be extended to take account of a probability mass at zero. We find that though ERp is robust to small proportions of zeros, XERp is more appropriate when these proportions become larger. We explore the impact of having to estimate p-values as the true proportion of zeros is unknown. We also outline the XERp software accompanying this paper. The Results & Discussion section demonstrates the sensitivity of the null distributions to the proportion of zeros via simulation and reports the bias and power associated with various p-value estimates. Next, we demonstrate XERp by applying it to a GC-MS metabolomics dataset. The experimental group represents patients suffering from tuberculosis meningitis (TBM) – a disease which is not expressed through one or more dominant diagnostic biomarkers. We find that XERp is able to select biologically relevant metabolites by extracting information from the frequency of zeros, as well as from the distributional shift. In addition, XERp retains the classification ability of ERp and performs well for new subjects, as well as in a leave-one-out (LOO) cross-validation context. We also discuss a comparison to imputation with non-zero values reported in the Additional file 2. Finally, we discuss the utility and future prospects of XERp.

## Methods

### Notation, terminology & null distribution assumptions

ERp, introduced in [14], aims to identify variables with significantly higher (upward shift) or lower (downward shift) values in the experimental group relative to the control group. Upward and downward classification rules are constructed based on a threshold value. The rates of misclassification for both shift directions are minimised over the thresholds, resulting in two minimised error rates ${\widehat{er}}_{up\;}^{*}$ and ${\widehat{er}}_{down\;}^{*}$ for each variable. These minimised error rates are then used as test statistics to test the hypothesis that the distribution of the variable is the same for the control and experimental groups, while the associated minimising thresholds are used to classify new subjects. ERp assumes a common continuous strictly increasing CDF under the null hypothesis. This does not cater for the possibility that the underlying variable assumes the value zero with positive probability. Here we extend the notation and terminology used in [14] to account for such a jump in the CDF at zero.

More specifically, consider a variable $W\ge 0$. It may be that the relevant metabolite is not present in the biological sample in which case $W=0$. It may also be that there is a detection limit $\delta >0$ and if $W\le \delta$ then the exact value of $W$ is unknown and the value 0 is recorded instead, while if $W>\delta$ then the exact value of $W$ is recorded. Calling the actually recorded variable $X$, it is related to the underlying variable $W$ by the equations $X=0$ if $W\le \delta$ and $X= W$ if $W>\delta$.

Denote the population CDF of $W$ by $H$ and let $H$ take the value ${\pi}^{*}$ in the point 0 (i.e. $H(0)={\pi}^{*}$) and assume that $H(x)$ is continuous and increasing in $x$ for $x\ge 0$, where $x$ denotes the argument at which the CDF is evaluated. The jump ${\pi}^{*}$ at 0 caters for the possibility that the underlying variable may take the value 0 with positive probability, representing instances where the metabolite is not present at all. Using the relation between the underlying variable $W$ and the observed variable $X$, it follows that the CDF of $X$ is given by $F(x)=\pi = H\left(\delta \right)$ for $0\le x\le \delta$ and $F(x)= H(x)$ for $x>\delta$. Notice that $\pi \ge \pi *$ since $\pi$ accounts for both the possibilities that the metabolite is not present and that it may be positive but below the detection limit. Next, let $G$ denote the conditional CDF of $X$ given that $X>0$, i.e. the positive part of the CDF of $X$. Formally,

$$ G(x)= P\left( X\le x\left| X>0\right.\right)=\frac{P\left(0< X\le x\right)}{P\left( X>0\right)}=\frac{F(x)- F(0)}{1- F(0)}=\frac{F(x)-\pi}{1-\pi} $$

Taking into account that $F(x)=\pi$ for $0\le x\le \delta$:

1 $$ G(x)=0\kern0.5em  f o r\kern0.5em 0\le x\le \delta \kern0.5em  and\kern0.5em  G(x)=\frac{F(x)-\pi}{1-\pi}\kern0.5em  f o r\kern0.5em  x>\delta $$

Then $F$ can be expressed in terms of $G$ by:

2 $$ F(x)=\pi \kern0.75em  f o r\kern0.5em 0\le x\le \delta \kern0.5em  and\kern0.5em  F(x)=\pi +\left(1-\pi \right) G(x)\kern0.5em  f o r\kern0.5em  x>\delta $$

**Fig. 1:**
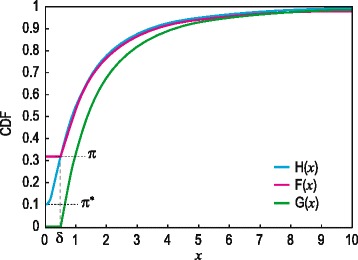
An illustration of the CDFs discussed. The graph illustrates the distributions of the variables $W$ and $X$ using a standard log-normal CDF to depict the positive part of $H(x)$, represented by the *blue line*, with ${\pi}^{*}$ set to 0.1. The assumptions on $H(x)$ imply that $G(x)$, represented by the *green line*, is continuous and increasing over $x>\delta$ with $\delta$ set to 0.5. For $x\le \delta$, $F(x)=\pi$ which is equal to 0.32 in this illustration, represented by the *pink line*. For $x>\delta$, $F(x)= H(x)$, but their graphs were slightly shifted for clarity

Figure 1 below illustrates these assumptions and notation. The CDF of $X$ is a mixture of a jump of size $\pi$ at $x=0$ and a continuous CDF $G(x)$ over $x>0$. Similar mixed distributions were used by Schisterman et al [16] in the context of estimating the Youden Index. In their estimation context, two distributions of this type are required, one for the control group and another for the experimental group. The XERp context is simpler in that only one such distribution is required since the distributions of the two groups are the same under the null hypothesis. XERp is also related to the developments of Ruopp et al. [17] for estimating the Youden Index in the presence of observations below the detection limit. However, XERp bases variable selection on the well-known concept of a p-value, providing the added benefit of a clear interpretation of the variable selection threshold (i.e. the significance level $\alpha$). The Youden Index, on the other hand, has no practical interpretation (as discussed in [14]). In the next section calculation of the null distributions of the error rate test statistics, required for conversion to p-values, are discussed in the XERp context.

### The null distribution of ${\widehat{er}}_{up\;}^{*}$

Denote the actual observed data by $\left({y}_n,{x}_n\right), n=1,2,\dots, N$. Here $N$ is the total number of subjects present and ${y}_n$ represents the group label of the $n$-th subject, taking the value 0 if a subject is in the control group or 1 if it is in the experimental group. Also ${x}_n$ represents the observed value of $X$ for the $n$-th subject. For the time being we restrict attention to the error rate associated with the upward rule. With $c\ge 0$ denoting a generic threshold, this rule classifies the $n$-th subject into the control group if ${x}_n\le c$ and into the experimental group otherwise. Let ${w}_0$ and ${w}_1$ (with ${w}_0 + {w}_1=1$) represent the respective relative costs of misclassification of control and experimental subjects. Then the error rate for the upward rule with threshold $c$ is

3 $$ {\widehat{er}}_{up}(c)=\frac{w_0}{N_0}{\displaystyle {\sum}_{n=1}^N I\left({y}_n=0,{x}_n> c\right)}+\frac{w_1}{N_1}{\displaystyle {\sum}_{n=1}^N I\left({y}_n=1,{x}_n\le c\right)} $$

Here ${N}_0$ and ${N}_1$ are the numbers of subjects in the control and experimental groups respectively. The minimised error rate is ${\widehat{er}}_{up\;}^{*}={min}_{c\ge 0}\left\{{\widehat{er}}_{up}(c)\right\}$ and this is still used as the test statistic to test the hypothesis that ${F}_0(x)={F}_1(x)= F(x)$, where $F(x)$ is the common CDF of $X$ under the null hypothesis for the control and experimental groups as in [14]. However, $F(x)$ must now take the form (2), requiring a revised calculation of the null distribution of ${\widehat{er}}_{up}^{*}$.

In the XERp context some ${x}_n$’s may be zero, while the non-zero ${x}_n$’s are all greater than the detection limit $\delta$. The event ${x}_n> c$ implies that ${x}_n$ is non-zero and therefore also that ${x}_n>\delta$. Hence (3) can be written as

4 $$ \begin{array}{l}{\widehat{er}}_{up}(c)=\kern0.5em \frac{w_0}{N_0}{\displaystyle {\sum}_{n=1}^N I\left({y}_n=0,{x}_n> c,{x}_n>\delta \right)}\\ {}+\kern0.5em \frac{w_1}{N_1}{\displaystyle {\sum}_{n=1}^N I\left({y}_n=1,{x}_n\le c,\ {x}_n>\delta \right)}+\frac{w_1}{N_1}{\displaystyle {\sum}_{n=1}^N I\left({y}_n=1,{x}_n\le c,{x}_n=0\right)}\end{array} $$

where we have split the second term according to the two events that ${x}_n>\delta$ and ${x}_n=0$. Next, evaluate each term in equation (4) for $c>\delta$ and $c\le \delta$. Considering the first term in (4), if $c>\delta$ the intersection of the events ${x}_n> c$ and ${x}_n>\delta$ is equivalent to ${x}_n> c$, which in turn is equivalent to $G\left({x}_n\right)> G(c)$. If $c\le \delta$ the intersection of the events ${x}_n> c$ and ${x}_n>\delta$ is equivalent to ${x}_n>\delta$, which in turn is equivalent to $G\left({x}_n\right)> G\left(\delta \right)= G(0)= G(c)$. Over all $c$, the intersection of the events ${x}_n> c$ and ${x}_n>\delta$ is thus equivalent to $G\left({x}_n\right)> G(c)$. Considering the second term in (4), if $c>\delta$ the intersection of the events ${x}_n\le c$ and ${x}_n>\delta$ is equivalent to $\delta <{x}_n\le c$, which in turn is equivalent to $0< G\left({x}_n\right)\le G(c)$. If $c\le \delta$ the intersection of ${x}_n\le c$ and ${x}_n>\delta$ is vacuous. Over all $c$, the intersection of the events ${x}_n\le c$ and ${x}_n>\delta$ is thus equivalent to $0< G\left({x}_n\right)\le G(c).$ Finally, consider the third term in (4), regardless of whether $c>\delta$ or $c\le \delta$, the intersection of the events ${x}_n\le c$ and ${x}_n=0$ is equivalent to ${x}_n=0$, which in turn is equivalent to $G\left({x}_n\right)= G(0)=0$. Equation (4) therefore reduces to:

5 $$ \begin{array}{l}{\widehat{er}}_{up}(c)=\frac{w_0}{N_0}{\displaystyle {\sum}_{n=1}^N I\left({y}_n=0,\  G\left({x}_n\right)> G(c)\right)}\\ {}+\kern0.5em \frac{w_1}{N_1}{\displaystyle {\sum}_{n=1}^N I\left({y}_n=1,\ 0< G\left({x}_n\right)\le G(c)\right)} + \frac{w_1}{N_1}{\displaystyle {\sum}_{n=1}^N I\left({y}_n=1,\  G\left({x}_n\right)=0\ \right)}\ \end{array} $$

Putting *u*_*n*_ = *G*(*x*_*n*_) and *b* = *G*(*c*), it follows that ${\widehat{er}}_{up}(c)$ in (5) can be restated as:

6 $$ \begin{array}{l}{\tilde{er}}_{u p}(b)=\frac{w_0}{N_0}{\displaystyle {\sum}_{n=1}^N I\left({y}_n=0,{u}_n> b\right)}+\frac{w_1}{N_1}{\displaystyle {\sum}_{n=1}^N I\left({y}_n=1,\ 0<{u}_n\le b\ \right)}\\ {}+\kern0.5em \frac{w_1}{N_1}{\displaystyle {\sum}_{n=1}^N I\left({y}_n=1,{u}_n=0\ \right)}\end{array} $$

Note that the terms in equation (6) neatly address the group differences we want to investigate. The first two terms evaluate the presence of an upward shift in the distribution of the experimental group, while the third term evaluates the presence of a difference in the proportion of zero observations.

The range $c\ge 0$ is equivalent to $0\le b\le 1$ so that ${\widehat{er}}_{up\;}^{*}={min}_{0\le b\le 1}\left\{{\widetilde{er}}_{up}(b)\right\}$. To derive the null distribution of ${\widehat{er}}_{up\;}^{*}$from this expression requires the common CDF of the ${u}_n$‘s. Note that the ${u}_n$‘s are independent and identically distributed ($i i d$), since the ${x}_n$‘s were assumed to be $i i d$ for the purpose of calculating the null distribution. By definition the common CDF of the ${u}_n$‘s is $P\left({u}_n\le u\right)$, where $u$ denotes the argument of the CDF. Considering separately the cases $u=0$ (equation 7) and $u>0$ (equation 8):

7 $$ P\left({u}_n=0\right)= P\left( G\left({x}_n\right)=0\right)= P\left({x}_n=0\right) = F(0)=\pi $$

8 $$ P\left({u}_n\le u\right)= P\left( G\left({x}_n\right)\le u\right)= P\left({x}_n\le {G}^{-1}(u)\right)= F\left({G}^{-1}(u)\right)=\pi +\left(1-\pi \right) G\left({G}^{-1}(u)\right)=\pi +\left(1-\pi \right) u $$

The common CDF of the ${u}_n$‘s is also a mixture between a jump at zero of size $\pi$ and a uniform distribution on (0,1). The distribution of the ${u}_n$ ‘s only depends on $\pi$ and, since ${\widetilde{er}}_{up}(b)$ is only a function of the ${u}_n$‘s, the same holds for ${\widehat{er}}_{up\;}^{*}={min}_{0\le b\le 1}\left\{{\widetilde{er}}_{up}(b)\right\}$.

**Fig. 2:**
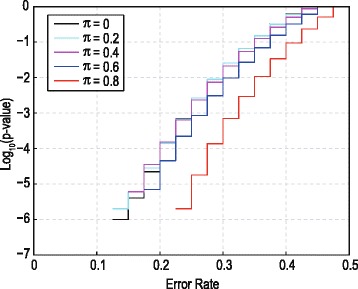
Null CDF for ${\widehat{er}}_{up\;}^{*}$ with $\pi$ taking on different values. One million simulation repetitions, group sizes ${N}_0={N}_1=20$ and the weight set ${w}_0={w}_1=\frac{1}{2}$ were used to calculate the null-distributions. Each line represents the CDF for a different value of $\pi$, $\pi =0$ (*black*), $\pi =0.2$ (*light blue*), $\pi =0.4$ (*purple*), $\pi =0.6$ (*dark blue*) and $\pi =0.8$ (*red*). The CDFs are plotted on a log_10_ scale for clarity purposes, since interest centres on the extreme left tail

To conclude, the null distribution of ${\widehat{er}}_{up\;}^{*}$ (equation 3) depends only on the parameter $\pi$ and not on any of the other parameters (${\pi}^{*}$, $\delta$ or the positive part CDF $G(x)$). Though ${\widehat{er}}_{up\;}^{*}$ is no longer fully non-parametric, as was the case in [14], the only remaining unknown parameter is $\pi$. Not needing to take into account ${\pi}^{*}$, $\delta$ or $G(x)$ when calculating p-values is a major advantage since these quantities are typically all unknown. Furthermore, the dependence on $\pi$ only becomes pronounced for larger $\pi$, as shown in Fig. 2 below. To calculate the null-distribution of ${\widehat{er}}_{up\;}^{*}$ for any given value of the parameter $\pi$ via simulation, the algorithm in Table 1 of [14] can still be followed, with the exception that the ${u}_n$‘s must be sampled from the CDF given by (7) and (8). This is easily achieved by drawing ${v}_n$ from a uniform (0,1) distribution and setting ${u}_n=0$ if ${v}_n\le \pi$ and ${u}_n=\left({v}_n-\pi \right)/\left(1-\pi \right)$ otherwise (refer to Section S2 of the Additional file 1 for more detail). In the next section, several solutions are proposed to finding p-values in the presence of the remaining unknown parameter $\pi$.

**Table 1:** XERp Results for TBM vs Healthy Controls

Variable	ER	C	Direction	Observed p-value	Percentage zeros		
					Control	Experimental	Overall
2-hydroxybutyric acid	0.08	0.3	Up	0	68%	0%	44%
3-hydroxyisovaleric acid	0.09	20.05	Up	0	0%	0%	0%
4-hydroxyphenylpyruvic acid	0.11	0	Up	0	100%	18%	71%
methylcitric acid	0.1	0.45	Up	0	55%	6%	38%
quinolinic acid	0.05	2.38	Up	0	45%	0%	29%
2-hydroxyvaleric acid	0.12	0	Up	0	77%	6%	52%
non-annotated-1	0.15	0	Up	0	100%	24%	73%
uracil	0.13	0.67	Up	0	48%	0%	31%
1,4-dihydroxycyclohexane	0.14	0.23	Up	0	55%	6%	38%
non-annotated-3	0.19	0	Up	0	100%	29%	75%
2-ketoglutaric acid	0.16	0.56	Up	0	55%	6%	38%
phenylacetylglutamine	0.17	1.02	Up	0	90%	24%	67%
hexanoic acid	0.16	0.34	Down	0	35%	35%	35%
pyruvic acid	0.17	0.25	Up	0	29%	0%	19%
isocitric acid	0.17	1.6	Up	0	32%	0%	21%
glycolic acid	0.17	12.98	Up	0	0%	0%	0%
pyroglutamic acid	0.17	4.69	Up	0	52%	18%	40%
vanillylmandelic acid	0.18	7.5	Up	0	13%	0%	8%

The table lists the discriminatory variables based on their significant ${p}_{obs}$-values when compared to B-H adjusted significance level ($\alpha =0.05$). The first column lists the names of these variables (labelled “Variable”), the second the minimized error rate (labelled “ER”) and the third the associated threshold (labelled “C”). The directions of the minimized error rates are given in the fourth column labelled “Direction”. Column five contains the ${p}_{obs}$-values. The last three columns also report the percentages of zeros in each group and in the combined data set

A simulation study was performed to assess the sensitivity of the null distribution of ${\widehat{er}}_{up\;}^{*}$ to changing values of $\pi$. Figure 2 illustrates these null distributions for $\pi$ varying from 0 to 0.8 in steps of 0.2. Note that the distributions are discrete, with jumps at the possible values of ${\widehat{er}}_{up}^{*}$, being the different numbers in the list $\left\{\left({w}_0{n}_0+{w}_1{n}_1\right)/ N:{n}_0=1,2,\dots, {N}_0;{n}_1=1,2,\dots, {N}_1\right\}$. The null distribution of ${\widehat{er}}_{up}^{*}$ changes slowly when $\pi$ is small - in fact the graphs for $\pi =0$ and 0.2 are almost indistinguishable in Fig. 2. Even at $\pi =0.4$ the differences are quite small, but become increasingly pronounced for larger $\pi$. Note that for $\pi =0.8$ the million simulation repetitions yielded no error rate values below 0.225, implying that the probability below 0.225 rapidly becomes very small for the sample sizes used in this illustration. In fact, under the null hypothesis the probability of the event ${\widehat{er}}_{up\;}^{*}=0$ can be calculated analytically. This is shown in Section S3 of the Additional file 1, which also gives this probability as a function of ${N}_0$, ${N}_1$ and $\pi$ in a reference table.

### Computing p-values

Next p-values need to be computed for observed values of ${\widehat{er}}_{up\;}^{*}$. This is no longer straightforward, since the null distribution depends on $\pi$ and we do not know its true value. In this section three possible choices of p-values are discussed.

Let ${p}_{\pi}$ denote the p-value of an observed ${\widehat{er}}_{up}^{*}$ when referred to the null CDF with the true parameter $\pi$. Since we do not actually know the value of $\pi$, we cannot use ${p}_{\pi}$ in practice and need specific choices. The first choice is ${p}_0$, which implies referring ${\widehat{er}}_{up}^{*}$ to the null CDF with $\pi =0$. This amounts to reverting back to the original ERp p-value and ignoring the possible effects of the zeros.

The second choice is the maximum p-value, defined as ${p}_{max}={max}_{0\le \pi \le 1}\left\{{p}_{\pi}\right\}$, and is aimed at ensuring the Type I error rate is controlled since rejection of the null hypothesis at significance level $\alpha$ using ${p}_{max}$ implies ${p}_{max}\le \alpha$. Therefore ${p}_{\pi}\le {p}_{max}\le \alpha$ and the null hypothesis will also be rejected if the true ${p}_{\pi}$ were used. The calculation of this estimate requires some additional simulation: (i) calculate the null distributions over a grid of all possible $\pi$ values given the sample sizes in each group (i.e. ${N}_0$ and ${N}_1$); (ii) list the individual p-values, pertaining to the observed error rate, as $\pi$ varies over this grid; (iii) then ${p}_{max}$ is the maximum in this list. In practice it is not necessary to use a fine grid since the maximum usually occurs at small choices of $\pi$ where the null-distribution does not change dramatically (Fig. 2).

The third choice estimates $\pi$ by the proportion of zeros observed in the dataset (denoted by $\widehat{\pi}$) and uses the corresponding p-value, i.e. ${p}_{obs}={p}_{\widehat{\pi}}$.

In datasets with a low frequency of zeros (reflected in $\widehat{\pi}$ being small), ${p}_0$, ${p}_{max}$ and ${p}_{obs}$ should yield similar results since the null distribution changes only slowly for small values of $\pi$ (Fig. 2). If one is determined to control the Type I error rate, ${p}_{max}$ would be a reasonable choice, but this may also imply loss of power. To investigate this further a comparative study was performed between the three proposed p-values, the details of which are reported in the Results & Discussion section. The outcomes of this investigation lead to our recommendation of ${p}_{obs}$ as the best choice.

### Other error rates

The developments and results discussed above are based on the upward rule error rate test statistic ${\widehat{er}}_{up}^{*}$, but can easily be extended to the downward and minimum error rates, ${\widehat{er}}_{down\;}^{*}$ and ${\widehat{er}}_{min\;}^{*}$, as described in more detail in Section S1 of the Additional file 1. Importantly, the null distributions of ${\widehat{er}}_{up\;}^{*}$ and ${\widehat{er}}_{down\;}^{*}$ are no longer the same if $\pi >0$, as was the case in [14].

### XERp software

XERp was programmed in Matlab [18] and all scripts and functions are provided as Additional file 2 along with an example application. The software allows the user to (i) generate the null distributions; (ii) rank variables based on XERp p-values; (iii) select variables for any given significance level after correcting for multiple testing by controlling either the family-wise error rate or the false discovery rate; and (iv) predict group membership of new samples or perform leave-one-out cross-validation. A discussion of the software, as well as a description of the results produced, are included in Section S4 of the Additional file 1, with a graphical overview provided as Additional file 1: Figure S1.

## Results & discussion

### Comparison of the p-values

Here we report the results of a comparative simulation study to assess the bias and power of the three p-value alternatives. Five performance metrics were used. Firstly, to assess estimation accuracy, the bias and mean squared error (MSE) were used. The bias and MSE were calculated by comparing the different p-value alternatives to ${p}_{\pi}$, conditioning on a 10% significance level. Next, the test size (referred to as the size) was used to assess the Type I error probability. The size represents the fraction of times the estimate falsely rejected the null hypothesis given a 10% significance level (i.e. $\alpha =0.1$). These three metrics were used to evaluate the performance of ${p}_0$, ${p}_{max}$ and ${p}_{obs}$ under the null hypothesis. Finally, the performance of the three p-values were also assessed under the alternative hypothesis. To do so the average p-value and the proportion of null hypothesis rejections (reported in the Additional file 1: Figure S4 and S5) were used to assess the discriminatory power of the different p-values. The alternative hypothesis was simulated using a log-normal (0,1) distribution for the control group and a shifted log-normal ($\mu$,1) distribution, with shift equal to $\mu$, for the experimental group. A jump component was added to these distributions by assuming different proportions of zeros in each group. The simulations were repeated a hundred thousand times for each shift. Null distributions were based on one million repetitions. A more detailed description of the steps to calculate all five performance metrics is provided in Section S5 and S6 of the Additional file 1. Two group size and weight scenarios were used. The first (scenario 1) corresponds to equal group sizes (${N}_0={N}_1=20$) and weights (${w}_0={w}_1=\frac{1}{2}$), while the second (scenario 2) is motivated by the metabolomics dataset used to illustrate XERp in Section 3.2, namely, ${N}_0=31;{N}_1=17;{w}_0=0.35;{w}_1=0.65$.

**Fig. 3:**
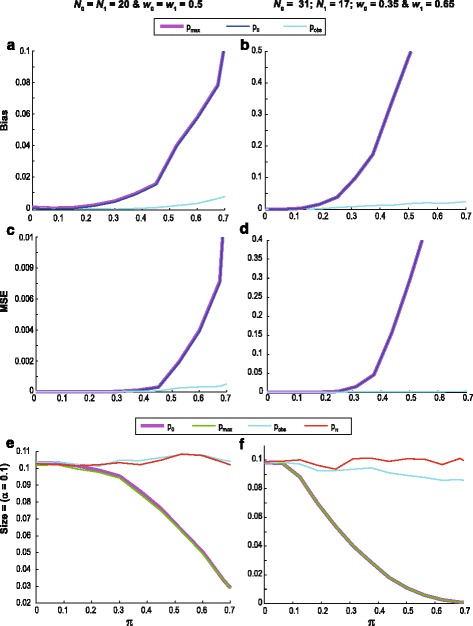
Bias, MSE and size of the three p-value alternatives for ${\widehat{er}}_{up\;}^{*}$. The number of simulation repetitions was a hundred thousand with $\pi$ varying from 0 to 0.7 and the group size and weigh choices as indicated (*left panel*: ${N}_0={N}_1=20$and${w}_0={w}_1=0.5$ and *right panel*: ${N}_0=31;{N}_1=17$and${w}_0=0.35;{w}_1=0.65$). The *lines* represent the results for the three p-value (i) ${p}_0$ in *dark blue*; (ii) ${p}_{max}$ in *purple*; and (iii) ${p}_{obs}$ in *light blue*. The *top row* depicts the bias, while the *middle row* depicts the MSE. The *red line* in the size graphs (*bottom row*) corresponds to ${p}_{\pi}$. The size was calculated for a significance level of 10%

Figure 3 shows graphs of the bias, MSE and size as functions of $\pi$ for scenarios 1 and 2. The results for ${p}_0$ and ${p}_{max}$ differ so little that they are almost indistinguishable. Moreover, they have rapidly increasing bias and MSE for increasing values of $\pi$ so that they are not recommendable unless one is quite certain that $\pi$ is small. The p-value alternative ${p}_{obs}$ does much better, having small bias and MSE, while remaining so for increasing values of $\pi$. Note that the bias and MSE graphs for ${p}_0$ and ${p}_{max}$ were truncated to make the bias and MSE of ${p}_{obs}$ more visible. The size results, given a 10% significance level, are presented in the final row of Fig. 3. Additional size results, given significance levels of 1 and 5%, are reported in Additional file 1: Figure S2. It is evident that ${p}_{obs}$ is better able to retain the significance level specified, compared to the other estimates regardless of whether $\pi$ is small or large.

Next, the performance of the three p-value alternatives is compared under the alternative hypothesis, i.e. there is either a difference in the proportion of zeros (the jump part) or in the continuous part of the distributions or in both. Define ${\pi}_0$ and ${\pi}_1$ as the population proportions of zeros among control experimental subjects respectively. The power was assessed given three ${\pi}_0$ and ${\pi}_1$ combinations. The first two represent instances where the proportion of zeros: (i) contains discriminatory information coinciding with the distributional shift (i.e. consonant variables where the group with the larger proportion of zeros has a lower mean [10, 12, 15]) represented by the choice ${\pi}_0=0.25;{\pi}_1=0$; and (ii) does not contain discriminatory information in that they are equal, represented by the choice ${\pi}_0={\pi}_1=0.25$. A third combination is included in the (Additional file 1: Figure S3) to evaluate the power given dissonant variables (i.e. where the proportion of zeros contains discriminatory information different from the distributional shift [10, 12, 15]) represented by the choice ${\pi}_0=0;{\pi}_1=0.25$. Though this is an unusual scenario (i.e. where the group with the larger proportion of zeros also has a higher mean) it is not unheard of as subjects react differently in the presence of disease and some diseases are known to cause different metabolic changes at different stages.

**Fig. 4:**
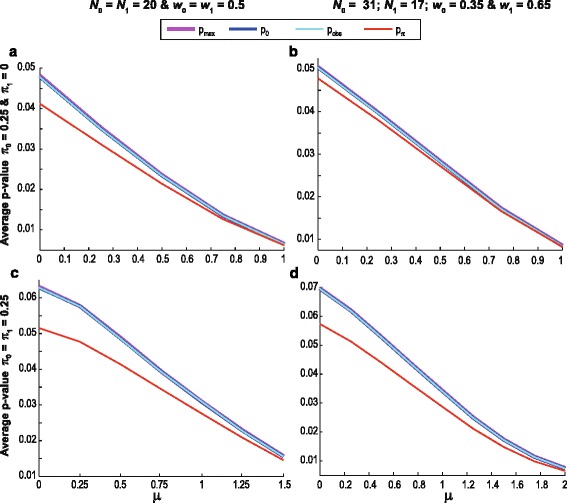
Measures of power of the three p-value alternatives for ${\widehat{er}}_{up\;}^{*}$. Observations for control subjects followed a log-normal (0,1) distribution while those of experimental subjects where drawn from a log-normal ($\mu$,1) distribution where $\mu$ assumed values ranging between 0 and 2. The number of simulation repetitions was a hundred thousand with ${\pi}_0$ and ${\pi}_1$ pairs selected as indicated (i.e. the *top row* ${\pi}_0=0.25;{\pi}_1=0$ and the *bottom row* ${\pi}_0={\pi}_1=0.25$). The graphs represent the two group size and weigh scenarios as indicated (*left panel*: ${N}_0={N}_1=20;{w}_0={w}_1=0.5$ and *right panel*: ${N}_0=31;{N}_1=17;{w}_0=0.35;{w}_1=0.65$). The *lines* represent the averages of the three p-value estimates (i) ${p}_0$ in *dark blue*; (ii) ${p}_{max}$ in *purple*; and (iii) ${p}_{obs}$ in *light blue*, conditioned on a 10% significance level. The *red lines* correspond to ${p}_{\pi}$

Figure 4 shows the average p-value as a measure of testing power for different zero proportions in the two groups and increasing distributional shifts in the experimental group $\left(\mu \right)$. To make the differences between the p-value alternatives more visible the average p-value was calculated conditioning on $p\le \alpha$ with $\alpha$ set to 10%. For the same reason the graphs are not displayed for the entire range of shift values ($\mu$). Once no differences between p-value alternatives are apparent and the average p-values have achieved sufficiently low levels the graphs are no longer displayed. The graphs comparing the proportions of null hypothesis rejections can be found in the Additional file 2. To interpret Fig. 4, it is important to note that when comparing p-value alternatives, the p-value with the lower average is able to detect difference faster given the conditions specified, i.e. it has greater power or smaller Type II errors. We find that the three p-value alternatives are almost indistinguishable in most instances and when differences are noted, ${p}_{obs}$ always outperforms ${p}_{max}$ and ${p}_0$. The differences between the three p-value alternatives and ${p}_{\pi}$ show the price paid in terms of power for not knowing the true value of $\pi$. On the basis of the evidence presented in Figs. 3 and 4, ${p}_{obs}$ is a better choice compared to${p}_0$ and ${p}_{max}$. The p-value recommended for use in XERp is therefore ${p}_{obs}$.

### Comparison to imputation

As a further assessment of XERp, a limited comparison to imputation was done. We only briefly discuss the setup and outcome of the comparison here, but report it in greater detail in Section S7 of the Additional file 1. The results from XERp, applied to the data with zeros, was compared to the results from ERp applied to the same data, but with zeros values replaced by positive random numbers. We find that replacing zeros with numbers imputed between some lower bound and the smallest non-zero value can have unwanted effects without real gains. The grid of classification thresholds evaluated, the minimum error rate statistic and corresponding minimising classification threshold and ultimately the associated p-value all become random to some extent when imputing zero values. If the p-value happens to be in the critical area an important variable may be missed or an unimportant variable may be selected by mistake, all depending on numbers chosen at random. Moreover, if a new subject has a zero value for a selected variable, the imputation rule used must first be applied. Classification is then based on whether this imputed number is larger or smaller than the threshold which may also have been estimated from imputed numbers. All this adds to unnecessary uncertainty about conclusions drawn without any obvious gains compared to simply using XERp.

### Application to metabolomics data

Here we report the results of XERp as applied to data generated from the GC-MS organic acid analysis of urine samples. Refer to the paper and SI of Mason et al. [19] for a full description of the processing of the data and clinical profiles of patients and controls. The dataset contains concentration levels for 185 variables observed in one experimental and two control groups. Variables refer to 180 substances that could be unequivocally annotated as metabolites and five with insufficient analytical-chemical information to be identified as metabolites, and thus designated as non-annotated variables. The experimental group consisted of 17 children diagnosed with TBM, referred to as the TBM group. The first control group consisted of 31 healthy infants, referred to as the Healthy Controls group. The second control group consisted of 21 seriously ill children whose initial clinical presentation was similar to the TBM cases, but subsequently proved to be negative for TBM, referred to as the Sick Controls group.

The results reported here firstly show the list of metabolites identified by XERp as important based on their ability to discriminate between TBM and Healthy Controls. The weight pair was used to adjust for the differences in sample size by setting them proportional to the inverses of the group sizes which leads to ${w}_0=\frac{N_1}{N_0+{N}_1}$ and ${w}_1=\frac{N_0}{N_0+{N}_1}$ when taking into account that they must add up to 1. One million simulation repetitions were performed to build the null distributions for the different comparisons, while ${\widehat{er}}_{min\;}^{*}$ was used as test statistic.

Table 1 shows selected output from the XERp software (as describe in the Additional file 2) for the metabolites selected based on${p}_{obs}$. The variable selection cut-off was based on the Bonferroni-Holm (B-H) multiple significance testing method, as explained in [14], at a family-wise error rate of 5%. Note that the software accompanying this paper can alternatively correct for multiple testing by controlling the false discovery rate, please refer to Section S4 of the Additional file 1 for more detail.

It is beyond the scope of this paper to provide an extensive interpretation of the biological significance of the eighteen variables listed in Table 1, but note that fifteen of the sixteen annotated metabolites bear some relationship to the clinical consequences in patients suffering from of TBM. The variables 2-hydroxybutyric, 3-hydroxyisovaleric, 4-hydroxyphenylpyruvic and 2-hydroxyvaleric acids are indicative of a perturbed energy metabolism in the patients due to the disease itself or as a consequence of antibiotic treatment. These variables are of low diagnostic value towards TBM. However, methylcitric acid most likely originated from the well-characterized methylcitrate cycle of *Mycobacterium tuberculosis* (*Mtb*), the bacterium known to induce TBM in the human host [20, 21]. The presence of quinolinic acid in the urine samples of the TBM patients is likely due to perturbations in the serotonin-tryptophane-pyridoxal phosphate pathways caused by TBM [22]. In accordance with these biological observations, methylcitric acid and quinolinic acid were recently proposed as two of four metabolites with high diagnostic potential for TBM [19]. Noteworthy: (1) A variable of unknown chemical structure (non-annotated-1) clearly highlights the importance of chemical characterization of unknown substances associated with infectious diseases in man, given the potential important diagnostic and translational value of these substances; (2) A gut metabolite, 4-hydroxyhipuric acid, included as an important indicator by Mason et al. [19], is not included in this list, but is selected when controlling the false discovery rate rather than the family-wise error rate.

Aside from the biological relevance of the variables listed in Table 1, we also note some valuable aspects of XERp. According to Table 1, 4-hydroxyphenylpyruvic acid contained 71% zero values overall of which 100% occurred in the control and 18% in the experimental group, while hexanoic acid contained 35% zero values in both groups. Both are listed as important by XERp, while both could just as easily have been excluded had zero-filtering been applied beforehand, even if the filter accounted for the group structure. Most importantly XERp is able to identify discriminatory variables regardless of whether $\pi$ is small (e.g. 3-hydroxyisovaleric acid with no zero observations) or large (e.g. non-annotated-3 with 75% zero observations) and without requiring any knowledge of the detection limit.

Secondly, the classification ability of the resulting list was assessed in two ways: (i) based on LOO cross-validation; and (ii) using the second control group as a hold-out set. This approach was followed for three reasons: (i) given the small group sizes we felt it unwise to select a hold-out or test set from the TBM and Healthy Control groups; (ii) small group sizes are common to metabolomics studies and as such it is important to make available and illustrate the LOO approach to assessing classification ability; and (iii) using a group of difficult to classify subjects (i.e. Sick Controls) allows us to assess the clinical practicality of the list, specifically, whether the list can distinguish between patients with TBM and patients with similar symptoms but not having TBM, indicated by the absence of *M*tb infection.

The LOO cross-validation results are reported in Table 2. In each iteration of the $N={N}_0+{N}_1$ LOO iterations, variables were selected based on p-values derived from the null CDF specific to the reduced group sizes and revised proportions of zeros. The CDFs were constructed using a hundred thousand simulation repetitions and the weight pairs did not change with the changing group sizes. Classification was performed in the exact same manner as described in [14]. We made use of the threshold resulting from the corresponding iteration to classify the subject left out. More details on how LOO cross-validation was performed are provided in the Additional file 2.

**Table 2:** LOO XERp Results for TBM vs Healthy Controls

Variable	% Selected	Classification Accuracy %			Average threshold	Direction
		Control Group	Experimental Group	Overall		
4-hydroxyphenylpyruvic acid	100	100	82	94	0	Up
non-annotated-1	100	100	76	92	0	Up
methylcitric acid	100	94	88	92	0.45	Up
quinolinic acid	100	87	94	90	2.38	Up
2-hydroxybutyric acid	100	77	94	83	0.31	Up
2-hydroxyvaleric acid	100	77	94	83	0	Up
phenylacetylglutamine	100	90	71	83	1.02	Up
3-hydroxyisovaleric acid	100	71	100	81	20.02	Up
1,4-dihydroxycyclohexane	100	71	88	77	0.23	Up
2-ketoglutaric acid	100	65	82	71	0.53	Up
uracil	100	61	88	71	0.69	Up
non-annotated-3	75	100	71	90	0	Up
hexanoic acid	67	65	94	75	0.35	Down
isocitric acid	33	52	94	67	1.67	Up
pyruvic acid	17	81	76	79	0.25	Up

The table displays an excerpt of the LOO XERp results. The table is sorted in descending order of the second column (“% Selected”), i.e. the percentage of times a variable (as listed in the first column under “Variable”) was selected out of the $N={N}_0+{N}_1$ LOO iterations. In addition, the table reports the specificity (accurate classification of control subjects) and sensitivity (accurate classification of experimental subjects) percentages in columns 3 and 4, as well as the overall classification accuracy in column 5. Columns 6 and 7 contain the threshold values averaged over all instances the variable was selected (“Average Threshold”) and the direction of the shift found to be significant (“Direction”)

Table 2 illustrates the variable selection stability of XERp, as is evident from the “% Selected” column. The top 11 variables (i.e. variables achieving a “% Selected” of 100) were consistently selected regardless of which subject was excluded. Notably, 10 of the top 11 correspond to the top 10 variables selected when no cross-validation is performed, with non-annotated-1, methylcitric and quinolinic acid in the top 4. The selection frequency of lower ranking variables dwindles quickly (75 to 17%), while the majority of variables were never selected (not shown). The classification accuracy of XERp is high with the top 9 variables achieving sensitivity and specificity levels exceeding 70% when classifying “left out” and therefore unseen subjects. The top 4 variables all had overall classification error rates (1-classification accuracy) of 10% or less, which may be a good argument to reduce the complexity of the list as a classification model to only 4. In addition, the average thresholds do not differ dramatically from those obtained using all available subjects (column 3 of Table 1), illustrating the robustness of XERp.

Table 3 contains the results when classifying the Sick Controls based on the variable list in Table 1. A prediction error is made when a Sick Control is classified into the TBM group. Interestingly non-annotated-3 made no classification errors, while non-annotated-1 only classified 3 Sick Controls as TBM and was also the second highest ranking variable in the LOO cross-validation. Though 4-hydroxyphenylpyruvic acid performed the best in the LOO cross-validation context, it is a marker of disease in general rather than a TBM-specific marker, as it only classified 62% of Sick Controls correctly.

**Table 3:** Classification Results for Sick Controls

Variables	Prediction Error	
	Count	Rate
non-annotated-3	0	0%
non-annotated-1	3	14%
phenylacetylglutamine	3	14%
pyroglutamic acid	3	14%
methylcitric acid	4	19%
2-hydroxyvaleric acid	7	33%
quinolinic acid	7	33%
3-hydroxyisovaleric acid	8	38%
4-hydroxyphenylpyruvic acid	8	38%
vanillylmandelic acid	9	43%
pyruvic acid	11	52%
1,4-dihydroxycyclohexane	12	57%
2-ketoglutaric acid	13	62%
glycolic acid	13	62%
uracil	13	62%
2-hydroxybutyric acid	16	76%
isocitric acid	16	76%
hexanoic acid	18	86%

The table lists the classification results for Sick Controls using variable selected when XERp is applied (without LOO cross-validation) to the TBM and Healthy Controls data. The first column lists the variable, while the second and third list the prediction error in terms of absolute count and percentage respectively

The potential diagnostic value of methylcitric acid and non-annotated variables was already discussed above. Phenylacetylglutamine and pyroglutamic acid also occurred in the list of important variables summarized in Table 1. Interesting, phenylacetylglutamine has been implicated in autism [23], while a metabolomics study highlighted pyroglutamic acid as one of 13 metabolites that differentiate between post-stroke patients group and healthy control subjects [24]. Both these neuropathological conditions are not related to TBM and indicate potential importance in clinical chemical studies of diseases that resemble meningitis, but are not caused by *Mtb*.

## Conclusion

We extended the ERp testing approach to take account of zeros occurring with positive probability by introducing a jump component into the CDF of the underlying variable and named it XERp. Though XERp is no longer nonparametric, it only requires the estimation of one parameter, the proportion of zeros, which can easily be estimated from the available data. XERp is able to simultaneously extract information from differences in the proportion of zeros between two groups as well as the distributional shifts. XERp does not require any knowledge of the detection limit. The most distinctive feature of XERp is that it is not only a variable selection tool, but also has the ability to directly classify new subjects.

XERp is favourable compared to combining ERp with random imputation of zero values. The latter may lead to threshold values that are only based on the random imputed values and therefore also would cause randomness in the classification of new subjects.

Future research will develop XERp in two ways. The two-part testing approaches briefly mentioned in the Background may hold some benefit as they are able to exploit both consonant as well as dissonant variables. Our first endeavour is therefore to develop XERp along the lines of a two-part test to ensure dissonant variables are correctly evaluated. Secondly, XERp is a univariate approach and only addresses the multivariate nature of metabolomics data by correcting for multiple testing. Future research will aim to generalize XERp to the multivariate setting.

## Additional files

Additional file 1:
**Figure S1**. Overview of the XERp software. **Figure S2**. Size of the three p-value alternatives. **Figure S3**. Average p-value (*p*≤0:1) for the dissonant case. **Figure S4**. Proportion of null hypothesis rejections given. *α* = 5%. **Figure S5**. Proportion of null hypothesis rejections given *α* = 1%. (PDF 532 kb)
Additional file 2: A compressed folder (XERp Software.zip) containing the Matlab scripts to perform XERp as well as an example application. (ZIP 11 kb)

## Abbreviations

B-H: Bonferroni-Holm multiple significance testing method
CDF: Cumulative distribution function
ERp: Variable selection for binary classification using error rate p-values
GC-MS: Gas chromatography-mass spectrometry
iid: Independently and identically distributed
LOO: Leave-one-out
MSE: Mean squared error
*Mtb*: Mycobacterium tuberculosis
SI: Supplementary information
XERp: Variable selection for binary classification based error rate p-values using an extension of ERp that accounts for zero.
