# Supplementary material for: Metabolomics variable selection and classification in the presence of observations below the detection limit using an extension of ERp
Source: BMC Bioinformatics. 2017 Feb 2;18:83. doi: 10.1186/s12859-017-1480-8 (PMC5290706; doi:10.1186/s12859-017-1480-8)
Supplement: Additional file 1: — Figure S1. Overview of the XERp software. Figure S2. Size of the three p-value alternatives. Figure S3. Average p-value (p≤0:1) for the dissonant case. Figure S4. Proportion of null hypothesis rejections given. α = 5%. Figure S5. Proportion of null hypothesis rejections given α = 1%. (PDF 532 kb) [file 12859_2017_1480_MOESM1_ESM.pdf]

Supplementary Information:

Metabolomics variable selection and classification in the presence of observations below the detection limit using an extension of ERp

Mari van Reenen[3,§], Johan A Westerhuis[1,3], Carolus J Reinecke[3], J Hendrik Venter[2]

[1]Biosystems Data Analysis, Swammerdam Institute for Life Sciences, University of Amsterdam, Science Park 904, 1098 XH Amsterdam, The Netherlands

[2]Centre for Business Mathematics and Informatics, Faculty of Natural Sciences, North-West University (Potchefstroom Campus), Private Bag X6001, Potchefstroom, South Africa.

[3]Centre for Human Metabolomics, Faculty of Natural Sciences, North-West University (Potchefstroom Campus), Private Bag X6001, Potchefstroom, South Africa

[§]Corresponding author

Email addresses:

MVR: 12791733@nwu.ac.za

JHV: hennie.venter@nwu.ac.za

JAW: J.A.Westerhuis@uva.nl

CJR: carools.reinecke@nwu.ac.za

Table of Content

S1. The null distribution of $\widehat{er}^*_{down}$ and $\widehat{er}^*_{min}$ p2

S2. Simulating the null distribution p4

S3. The null hypothesis probability of getting a zero error rate p5

S4. XERp Software p7

S5. Comparison of the p-values under the null hypothesis p10

S6. Comparison of the p-values under the alternative hypothesis p12

S7. Comparison to random imputation p17

References p19

S1. The null distribution of $\widehat{er}^*_{down}$ and $\widehat{er}^*_{min}$

Similar to the notation used in the main text, denote the actual observed data by (y_n, x_n) , $n = 1, 2, \ldots N$. Here y_n represents the group label of the n-th subject, taking the value 0 if it is in the control group and 1 if it is in the experimental group. Also x_n represents the observed value of X for the n-th subject. The definitions of minimized error rates for the down rule remains unchanged in the presence of zeros, i.e. $\widehat{er}^*_{down} = min_{c\geq 0}\{\widehat{er}_{down}(c)\}$ where:

$$\widehat{er}_{down}(c) = \frac{w_0}{N_0}\sum_{n=1}^{N} I(y_n = 0, x_n \leq c) + \frac{w_1}{N_1}\sum_{n=1}^{N} I(y_n = 1, x_n > c)$$

Again $F(x)$ is the common CDF of X under the null hypothesis for both the control and experimental groups, but $F(x)$ must now take the form (2) in the main paper. That is, some x_n's may be zero, while the non-zero x_n's are all greater than the detection limit $\delta > 0$:

$$\widehat{er}_{down}(c) = \frac{w_0}{N_0}\sum_{n=1}^{N} I(y_n = 0, x_n \leq c, x_n > \delta) + \frac{w_0}{N_0}\sum_{n=1}^{N} I(y_n = 0, x_n \leq c, x_n = 0) + \frac{w_1}{N_1}\sum_{n=1}^{N} I(y_n = 1, x_n > c, x_n > \delta) + \frac{w_1}{N_1}\sum_{n=1}^{N} I(y_n = 1, x_n > c, x_n = 0)$$

Now evaluate each term in the above equation for $c > \delta$ and $c \leq \delta$. Considering the first term, if $c > \delta$ the intersection of the events $x_n \leq c$ and $x_n > \delta$ is equivalent to $\delta < x_n \leq c$, which in turn is equivalent to $0 < G(x_n) \leq G(c)$. If $c \leq \delta$ the intersection of $x_n \leq c$ and $x_n > \delta$ is vacuous. Considering the second term, regardless of whether $c > \delta$ or $c \leq \delta$, the intersection of the events $x_n = 0$ and $x_n \leq c$ is equivalent to $x_n = 0$, which in turn is equivalent to $G(x_n) = G(0) = 0$. Consider the third term, if $c > \delta$ the intersection of the events $x_n > c$ and $x_n > \delta$ is equivalent to $x_n > c$, which in turn is equivalent to $G(x_n) > G(c)$. If $c \leq \delta$ the intersection of the events $x_n > c$ and $x_n > \delta$ is equivalent to $x_n > \delta$, which in turn is equivalent to $G(x_n) > G(\delta)$ or $G(x_n) > 0$. Consider the fourth term, regardless of whether $c > \delta$ or not, the intersection of the events $x_n > c$ and $x_n = 0$ is vacuous and this term is equal to zero.

Putting $u_n = G(x_n)$ and $b = G(c)$, it follows that $\widehat{er}_{down}(c)$ can be restated as:

$$\widehat{er}_{down}(b) = \frac{w_0}{N_0}\sum_{n=1}^{N} I(y_n = 0, 0 < u_n \leq b) + \frac{w_0}{N_0}\sum_{n=1}^{N} I(y_n = 0, u_n = 0\ )$$

$$+ \frac{w_1}{N_1}\sum_{n=1}^{N} I(y_n = 1, u_n > b\ \ )$$

The range $c \geq 0$ is equivalent $0 \leq b \leq 1$ so that $\widehat{er}^*_{down} = min_{0 \leq b \leq 1}\{\widehat{er}_{down}(b)\}$. To derive the null distribution of $\widehat{er}^*_{down}$ from this expression requires the common CDF of the u_n's. Note that the u_n's are independent and identically distributed (iid), since the x_n's were assumed to be iid for the purpose of calculating the null distribution. The common CDF of the u_n's, which by definition is equal to $P(u_n \leq u)$, was derived in the main text as $P(u_n \leq u) = \pi$ for $u = 0$ while $P(u_n \leq u) = \pi + (1 - \pi)u$ for $u > 0$. Again, the common distribution of the u_n's is also a mixture between a jump at zero of the size π and a uniform distribution on (0,1). The distribution of the u_n's only depends on π and since $\widehat{er}_{down}(b)$ is only a function of the u_n's, the same holds for $\widehat{er}^*_{down} = min_{0 \leq b \leq 1}\{\widehat{er}_{down}(b)\}$. To conclude, the null distribution of $\widehat{er}^*_{down}$ depends only on the parameter π and can be simulated in the same way as $\widehat{er}^*_{up}$. However, since the differences between the equation for $\widehat{er}_{up}(b)$ and $\widehat{er}_{down}(b)$ are no longer just in terms of the inequalities, i.e. $>$ vs $\geq$ and $<$ vs $\leq$ as described in [1], the null distributions are no longer the same.

Finally, the distribution of for the minimum rule can be obtained by simulating $\widehat{er}^*_{min} = min\left[min_b\{\widehat{er}_{up}(b)\}, min_b\{\widehat{er}_{down}(b)\}\right]$

S2. Simulating the null distribution

The original ERp approach required the following steps to simulate the null distributions needed to convert error rates (in this instance $\widehat{er}_{up}^{*}$) into p-values:

- Generate N independent observations from a uniform (0,1) distribution, i.e. the u_n's
- Assign the first N_0 $y_n's$ as 0 and the remainder as 1
- Minimize $\frac{w_0}{N_0}\sum_{n=1}^{N}(1-y_n)I(u_n > b) + \frac{w_1}{N_1}\sum_{n=1}^{N} y_n I(u_n \leq b)$ by varying b over the midpoints of the increasingly ordered u_n's to obtain $\widehat{er}_{up}^{*}$
- Repeat these steps M times to build up a file of iid copies of $\widehat{er}_{up}^{*}$, say $\widehat{er}_{up}^{*}(m),\ m = 1, \dots, M,$ providing a simulation approximation of the null CDF
- If T of the $\widehat{er}_{up}^{*}(m)$'s fall below an actually observed $\widehat{er}_{up}^{*}$ its associated p-value is approximately T/M. Approximations are more accurate for large M.

In the XERp context the u_n's must be sampled from the CDF given by (6a) and (6b), as explained in the main manuscript. We do so by first defining v_n's as iid observations from a uniform (0,1) distribution and express the u_n's relative to the v_n's by setting $u_n = 0$ if $v_n \leq \pi$ and $u_n = (v_n - \pi)/(1-\pi)$. To prove that this substitution has the desired effect, we again look at the common CDF of the u_n's (i.e. $P(u_n \leq u)$, where u denotes the argument of the CDF). Considering separately the cases $u = 0$ and $u > 0$

$$P(u_n = 0) = P(v_n \leq \pi) = \pi$$

and for $u > 0$

$$\begin{aligned} P(u_n \leq u) &= P(u_n \leq u, v_n \leq \pi) + P(u_n \leq u, v_n > \pi) \\ &= P(v_n \leq \pi) + P\left(\frac{v_n - \pi}{1-\pi} \leq u, v_n > \pi\right) \\ &= \pi + P(v_n \leq \pi + (1-\pi)u, v_n > \pi) \\ &= \pi + (1-\pi)u \end{aligned}$$

S3. The null hypothesis probability of getting a zero error rate

Here we provide an explicit formula for $P_0(\widehat{er}_{up}^* = 0)$. The event $\widehat{er}_{up}^* = 0$ happens if and only if all the x_n-values of the experimental group are all larger than those of the control group. Under the null hypothesis this amounts to all the u_n-values in (5) corresponding to the experimental group being larger than those corresponding to the control group. Under the null hypothesis, the u_n's are iid according to (6a) and (6b) and then

$$P_0(\widehat{er}_{up}^* = 0) = \int_0^1 [\pi + (1-\pi)u]^{N_0}[1-\pi-(1-\pi)u]^{N_1-1}N_1(1-\pi)du$$

$$= N_1(1-\pi)^{N_1}\sum_{i=0}^{N_0}\frac{N_0!}{i!\,(N_0-i)!}\pi^i(1-\pi)^{N_0-i}\int_0^1 u^{N_0-i}(1-u)^{N_1-1}\,du$$

$$= N_1(1-\pi)^{N_1}\sum_{i=0}^{N_0}\frac{N_0!}{i!\,(N_0-i)!}\pi^i(1-\pi)^{N-i}\frac{(N_0-i)!\,(N_1-1)!}{(N_0-i+N_1)!}$$

$$= N_0!\,N_1!\sum_{i=0}^{N_0}\frac{1}{i!\,(N-i)!}\pi^i(1-\pi)^{N-i}$$

This formula gives the null hypothesis probability for a zero upward rule error rate value. In case of a downward rule error rate the same formula applies but with the roles of N_0 and N_1 interchanged. For the minimum rule the two probabilities must be added. Below is a table which shows the upward rule zero probabilities for N_0 and N_1 ranging from 5 to and 10 and π varying from 0 to 0.9 in steps of 0.1. The zero probabilities decrease as π increases, slowly at first but then more rapidly. It also decreases with increasing sample sizes. For sample sizes both 10 or larger, the zero probability is below 0.000005 regardless of π. In particular, if all the control measurements are zero and all the experimental measurements are positive in such a case, then $\widehat{er}_{up}^* = 0$ and the corresponding exact p-value is below 0.000005.

		π									
N_0	N_1	0	0.1	0.2	0.3	0.4	0.5	0.6	0.7	0.8	0.9
5	5	0.003968	0.003968	0.003943	0.003780	0.003309	0.002472	0.001456	0.000596	0.000130	0.000006
5	6	0.002165	0.002164	0.002139	0.001995	0.001631	0.001082	0.000534	0.000169	0.000025	0.000001
5	7	0.001263	0.001262	0.001238	0.001114	0.000840	0.000489	0.000200	0.000049	0.000005	0.000000
5	8	0.000777	0.000776	0.000754	0.000648	0.000446	0.000226	0.000076	0.000014	0.000001	0.000000
5	9	0.000500	0.000499	0.000478	0.000390	0.000243	0.000106	0.000029	0.000004	0.000000	0.000000
5	10	0.000333	0.000332	0.000313	0.000240	0.000134	0.000050	0.000011	0.000001	0.000000	0.000000
6	5	0.002165	0.002164	0.002160	0.002118	0.001949	0.001571	0.001011	0.000455	0.000109	0.000006
6	6	0.001082	0.001082	0.001078	0.001040	0.000911	0.000663	0.000362	0.000128	0.000021	0.000001
6	7	0.000583	0.000583	0.000579	0.000546	0.000449	0.000291	0.000133	0.000036	0.000004	0.000000
6	8	0.000333	0.000333	0.000329	0.000302	0.000231	0.000132	0.000050	0.000010	0.000001	0.000000
6	9	0.000200	0.000200	0.000196	0.000174	0.000122	0.000061	0.000019	0.000003	0.000000	0.000000
6	10	0.000125	0.000125	0.000122	0.000103	0.000066	0.000028	0.000007	0.000001	0.000000	0.000000
7	5	0.001263	0.001263	0.001262	0.001251	0.001190	0.001018	0.000709	0.000349	0.000092	0.000005
7	6	0.000583	0.000583	0.000582	0.000572	0.000526	0.000413	0.000248	0.000096	0.000018	0.000001
7	7	0.000291	0.000291	0.000291	0.000282	0.000248	0.000176	0.000090	0.000027	0.000003	0.000000
7	8	0.000155	0.000155	0.000155	0.000148	0.000122	0.000078	0.000033	0.000008	0.000001	0.000000
7	9	0.000087	0.000087	0.000087	0.000081	0.000063	0.000035	0.000012	0.000002	0.000000	0.000000
7	10	0.000051	0.000051	0.000051	0.000046	0.000033	0.000016	0.000005	0.000001	0.000000	0.000000
8	5	0.000777	0.000777	0.000777	0.000774	0.000752	0.000673	0.000503	0.000269	0.000077	0.000005
8	6	0.000333	0.000333	0.000333	0.000330	0.000314	0.000262	0.000171	0.000073	0.000015	0.000000
8	7	0.000155	0.000155	0.000155	0.000153	0.000141	0.000108	0.000061	0.000020	0.000003	0.000000
8	8	0.000078	0.000078	0.000078	0.000076	0.000067	0.000046	0.000022	0.000006	0.000001	0.000000
8	9	0.000041	0.000041	0.000041	0.000039	0.000033	0.000021	0.000008	0.000002	0.000000	0.000000
8	10	0.000023	0.000023	0.000023	0.000021	0.000017	0.000009	0.000003	0.000000	0.000000	0.000000
9	5	0.000500	0.000500	0.000499	0.000499	0.000491	0.000455	0.000360	0.000208	0.000065	0.000005
9	6	0.000200	0.000200	0.000200	0.000199	0.000193	0.000170	0.000119	0.000056	0.000012	0.000000
9	7	0.000087	0.000087	0.000087	0.000087	0.000082	0.000068	0.000041	0.000015	0.000002	0.000000
9	8	0.000041	0.000041	0.000041	0.000041	0.000037	0.000028	0.000015	0.000004	0.000000	0.000000
9	9	0.000021	0.000021	0.000021	0.000020	0.000018	0.000012	0.000005	0.000001	0.000000	0.000000
9	10	0.000011	0.000011	0.000011	0.000010	0.000009	0.000005	0.000002	0.000000	0.000000	0.000000
10	5	0.000333	0.000333	0.000333	0.000333	0.000330	0.000313	0.000261	0.000161	0.000055	0.000004
10	6	0.000125	0.000125	0.000125	0.000125	0.000122	0.000112	0.000084	0.000042	0.000010	0.000000
10	7	0.000051	0.000051	0.000051	0.000051	0.000050	0.000043	0.000028	0.000012	0.000002	0.000000
10	8	0.000023	0.000023	0.000023	0.000023	0.000022	0.000017	0.000010	0.000003	0.000000	0.000000
10	9	0.000011	0.000011	0.000011	0.000011	0.000010	0.000007	0.000004	0.000001	0.000000	0.000000
10	10	0.000005	0.000005	0.000005	0.000005	0.000005	0.000003	0.000001	0.000000	0.000000	0.000000

Table S1. Null hypothesis probabilities of the event that $\widehat{er}^*_{up} = 0$

S4. XERp Software

Here we describe the XERp software, its variations, the inputs required and results produced. XERp was programmed in Matlab [2] and the scripts are provided in addition to this document of which Figure S1 provides a graphical overview. The following inputs are required:

- A data matrix containing the observed values for all subjects (represented by the rows of the matrix) and variables (represented by the columns of the matrix).
- The variable names in a separate column vector of type character and the same length as the second dimension of the data matrix (i.e. each variable must have a name).
- A vector containing the shift direction of interest for each variable and determining the test statistic to be used. The value 1 indicates that an upward shift should be evaluated using as a test statistic"; the value -1 indicates that a downward shift should be evaluated using as a test statistic"; and the value 0 indicates that both shift directions should be evaluated using as a test statistic".
- A categorical row vector indicating the group membership of each subjects. The vector must have the same length as the first dimension of the data matrix (i.e. each subject must have been assigned to a group).
- The two string variables used as group labels in the group membership vector.
- The cost of misclassification into the control group. The cost of misclassification into the experimental group is derived from the constraint on the weights, i.e. $w_0 + w_1 = 1$. Alternatively, the user can set $w_0 = 0$ which prompts the software to calculate the weights from the group sizes, as explained in the main text, i.e. $w_0 = \frac{N_1}{N_0+N_1}$ and $w_1 = \frac{N_0}{N_0+N_1}$
- The value of the preferred significance level α as a separate numeric input.
- The method to use when correcting for multiple testing as a separate string variable. There are two options (i) 'FDR' controls the false discovery rate and makes use of the fdr script of Arnaud Delorme (2008) sourced through MathWorks (https://uk.mathworks.com/matlabcentral/fileexchange/27960-resampling-statistical-toolkit/content/statistics/fdr.m). (ii) 'BH' controls the familywise error rate using the Bonferroni-Holm approach, as described in [1].
- The number of simulations to use when calculating the null distributions.
- The file names to use when saving and exporting results.

These user inputs must then be presented to the different scripts provided to (i) generate the null distributions; (ii) perform variable selection based on XERp; (iii) predict group membership based on the

results from the variable selection function; or (iv) perform leave-one-out (LOO) cross-validation. The scripts must be called in sequence as results files from one script are required as input to other scripts. The processing sequence is illustrated in Figure S1 as well as in the example provided (the Example.m file). In addition, the “help” command can also be used to ascertain the required inputs and resulting output of each script.

Note that a different data may be provided when performing prediction to allow for the classification of subjects other than those used to perform variable selection. Doing so requires a data matrix of observations for the same variables originally presented, but for the new subjects. If the group membership of the new subjects is known, this can also be provided to the prediction script (XERp_Predict.m) which will add this information to the results for easier interpretation. Further, all predictions are performed on a per variable basis and the final classification is left to the user. One way to achieve this is by simply using majority vote as explained in [1].

The results from the software are automatically exported to an Excel workbook with the name provided. Two sets of results can be generated, namely, the results when using all subjects (i.e. the full sample or FS results) and the results when applying leave-one-out cross-validation (i.e. the LOO results). The FS results workbook contains the variable selection and classification (i.e. group membership prediction) results. The LOO results workbook contains the results for each iteration, namely: (i) a binary indicator taking the value 1 if a variable was significant and selected as predictor; (ii) the minimizing threshold for each variable; (iii) the shift direction producing the smallest error rate, if the directional vector was set to 0, or the direction specified; and (iv) the group membership prediction for each variable for the subject “left out”. In addition, a summary of the LOO results is provided.

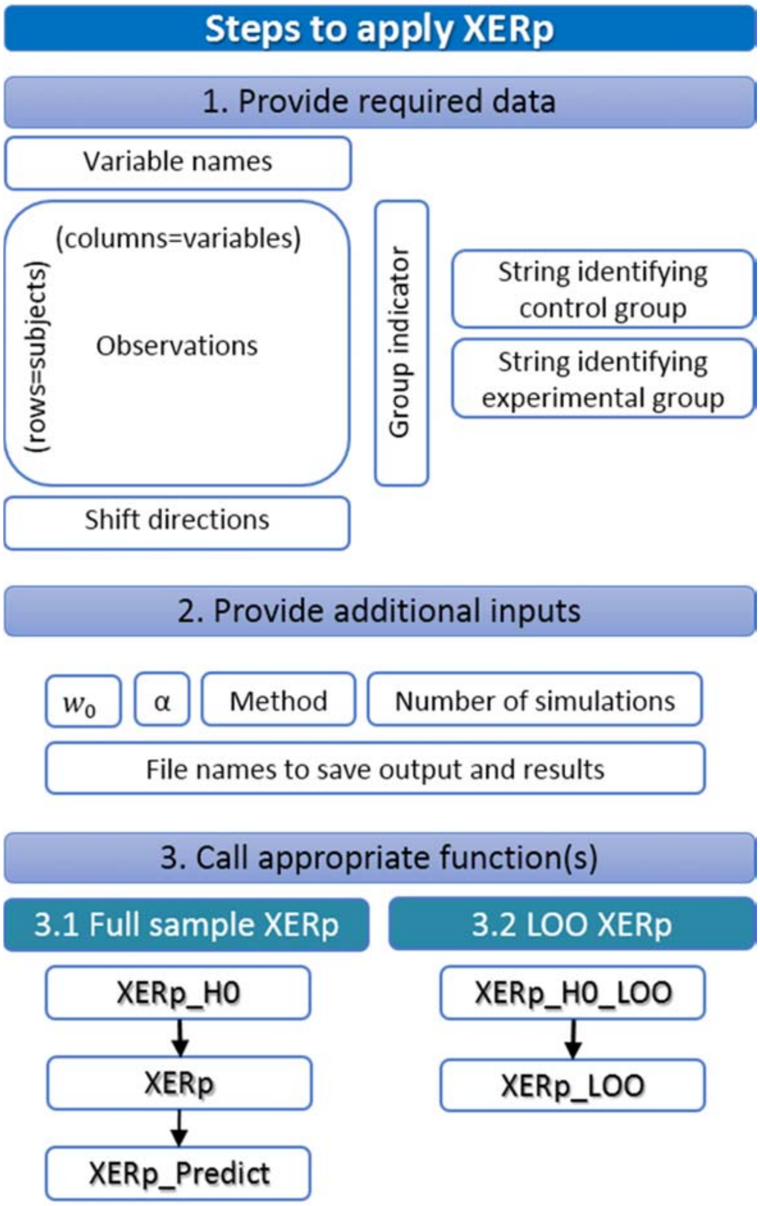

Figure S1. **Overview of the XERp software**

S5. Comparison of the p-values under the null hypothesis

A comparative simulation study to assess the bias and power of the three p-value alternatives was done as discussed in the main text. The detailed simulation steps used to evaluate the bias, MSE and size are as follows:

1. Choose values for N_0 , N_1 , w_0 and w_1.
2. Let $\{\pi_1, \pi_2, \dots, \pi_J\}$ denote a grid of π values. It is convenient to choose $J = N = N_0 + N_1$ and $\pi_j = (j-1)/J$ (guaranteeing a sufficient grid). For each π in the grid compute the null CDF by simulation and table the results, referred to as the CDF-Table below.
3. For $j = 1, 2, \dots, J$ and for $m = 1, 2, \dots, M$
   - 3.1. Generate $\{v_n(j,m),\ n = 1, 2, \dots, N\}$ iid from the uniform $(0,1)$ distribution and transform them to $u_n(j,m) = 0$ if $v_n(j,m) \le \pi_j$ and $u_n(j,m) = (v_n(j,m) - \pi_j)/(1 - \pi_j)$ otherwise.
   - 3.2. Put $y_n(j,m) = 0$ for $n = 1, 2, \dots, N_0$ and $y_n(j,m) = 1$ for $= N_0 + 1, N_0 + 2, \dots, N$.
   - 3.3. Compute $\widehat{er}_{up}^{*}(j,m)$ by minimizing (5) based on the data $\{y_n(j,m), u_n(j,m)\}$, $n = 1, 2, \dots, N$.
   - 3.4. Convert $\widehat{er}_{up}^{*}(j,m)$ to the three p-value alternatives as defined in the main text. Also convert $\widehat{er}_{up}^{*}(j,m)$ to the true p-value by referring the $\widehat{er}_{up}^{*}(j,m)$ to the column corresponding to π_j in the CDF-table, calling the result $p_{\pi_j}(j,m)$.
4. Finally, $B(j) = \frac{1}{M}\sum_{m=1}^{M}\{p'(j,m) - p_{\pi_j}(j,m)\}$; $MS(j) = \frac{1}{M}\sum_{m=1}^{M}\{p'(j,m) - p_{\pi_j}(j,m)\}^2$ and $S(j) = \frac{1}{M}\sum_{m=1}^{M} I(p'(j,m) \le p_{\pi_j}(j,m))$ estimates the bias, MSE and size of the three p-values (represented here in general by $p'(j,m)$), respectively, when π_j is the true value of π.

Figure S2 shows the test size (referred to as the size) used to assess the Type I error probability, as was done in the main text. Again, the size represents the fraction of times the estimate falsely rejected the null hypothesis, but for significance levels of 5 % (Figures S2a and S2b) and 1 % (Figures S2c and S2d). It is again evident that p_{obs} is better able to retain the significance level specified, compared to the other estimates regardless of whether π is small or large. The reader may note the seemingly unexpected instability of p_{π}, but this is simply due to the discrete nature of the distributions which become especially apparent in the tails.

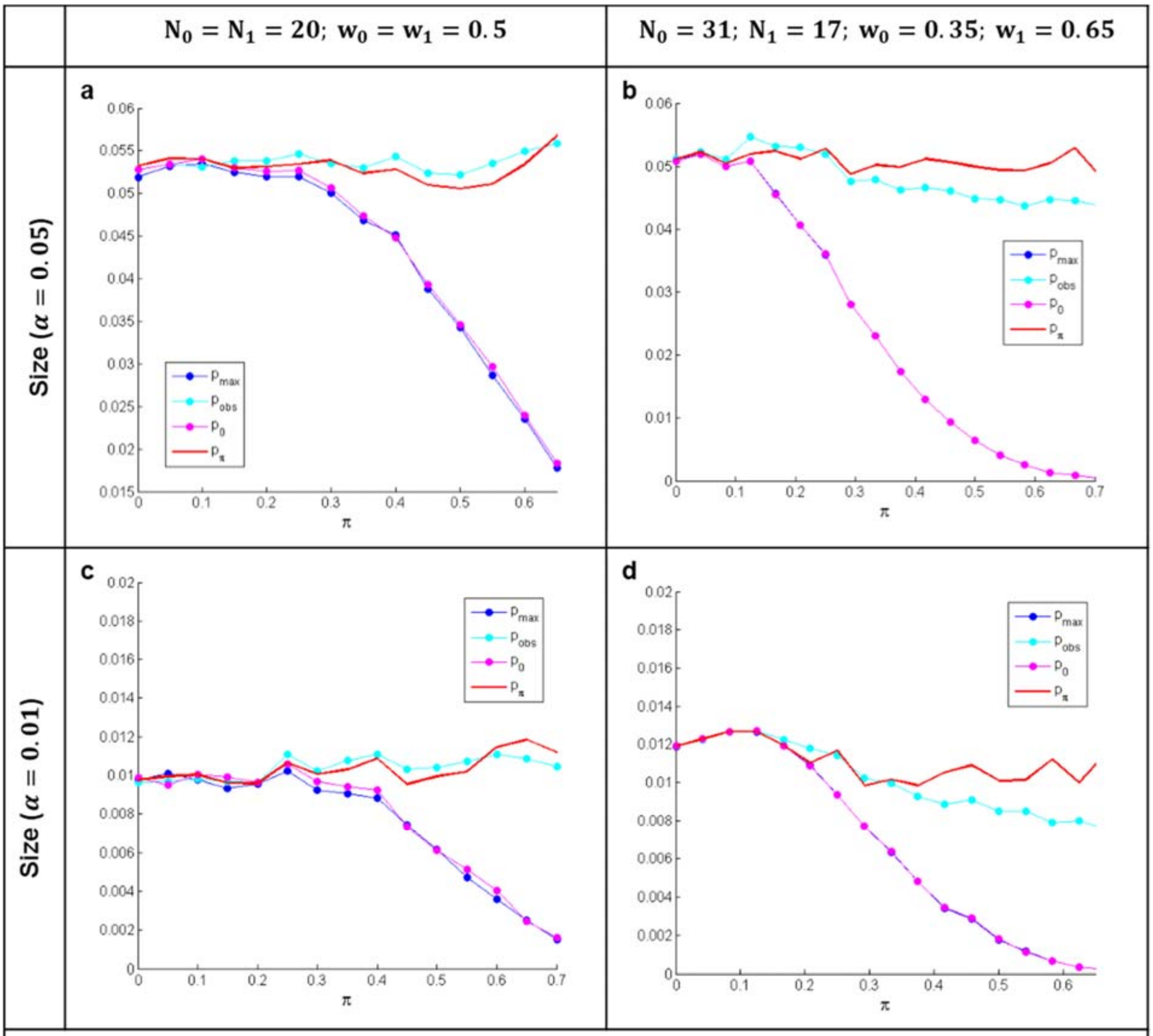

Figure S2 Size of the three p-value alternatives

S6. Comparison of the p-values under the alternative hypothesis

The comparative simulation study done to assess the power of the three p-value alternatives followed the following simulation steps:

1. Choose values for N_0 , N_1 , w_0 and w_1.

2. Choose a grid of distributional shift values $\{\mu_k,\ k = 1, 2, \dots, K\}$

3. For $k = 1, 2, \dots, K$ and for $m = 1, 2, \dots, M$

   3.1. Generate $\{X_n^0(k,m),\ n = 1, 2, \dots, N_0\}$ from the LN (0,1) distribution. Now, generate $\{v_n(k,m),\ n = 1, 2, \dots, N_0\}$ from the uniform (0,1) distribution and set $X_n^0(k,m) = 0$ if $v_n(k,m) \leq \pi_0$ and $X_n^0(k,m) = (X_n^0(k,m) - \pi_0)/(1 - \pi_o)$ otherwise The $X_n^0(k,m)$ represent the observations for the control group.

   3.2. Generate $\{X_n^1(k,m),\ n = 1, 2, \dots, N_1\}$ from the LN (0,μ_k) distribution. Now, generate $\{v_n(k,m),$ $n = 1, 2, \dots, N_1\}$ from the uniform (0,1) distribution and set $X_n^1(k,m) = 0$ if $v_n(k,m) \leq \pi_1$ and $X_n^1(k,m) = (X_n^1(k,m) - \pi_1)/(1 - \pi_1)$ otherwise The $X_n^1(k,m)$ represent the observations for the experimental group.

   3.3. Combine the $\{X_n^0(k,m),\ n = 1, 2, \dots, N_0\}$ and $\{X_n^1(k,m),\ n = 1, 2, \dots, N_1\}$ observations as $\{X_n(k,m),$ $n = 1, 2, \dots, N\}$ and put $y_n(k,m) = 0$ for $n = 1, 2, \dots, N_0$ and $y_n(j,m) = 1$ for $= N_0 + 1, N_0 +\ 2, \dots, N$.

   3.4. Compute $\widehat{er}_{up}^*\,(k,m)$ by minimizing (5) based on the data $\{y_n(k,m), X_n(k,m)\}$, $n = 1, 2, \dots, N$.

   3.5. Convert $\widehat{er}_{up}^*\,(k,m)$ to the three p-values as defined in the main text. Also convert $\widehat{er}_{up}^*\,(k,m)$ to the true p-value by referring the $\widehat{er}_{up}^*\,(k,m)$ to the column corresponding to $\pi = \frac{\pi_0 N_0 + \pi_1 N_1}{N_0 + N_1}$ in the CDF-table, calling the result $p_\pi(k,m)$.

4. Finally, $A(k) = \frac{1}{M}\sum_{m=1}^{M}\{p'(k,m)\}$ and $PR(j) = \frac{1}{M}\sum_{m=1}^{M} I(p'(k,m) \leq \alpha)$ estimates the average p-value and proportion of rejection for the three p-values, respectively, for a given significance level α.

Next we report further results on the performance of the p-value alternatives under the alternative hypothesis based on the average p-value and the proportion of null hypothesis rejections. The alternative hypothesis was simulated using a shifted log-normal (LN) distribution for the experimental groups compared to a LN(0,1) distribution for the control group. A jump component was added to these distributions by assuming different proportions of zero values in each group.

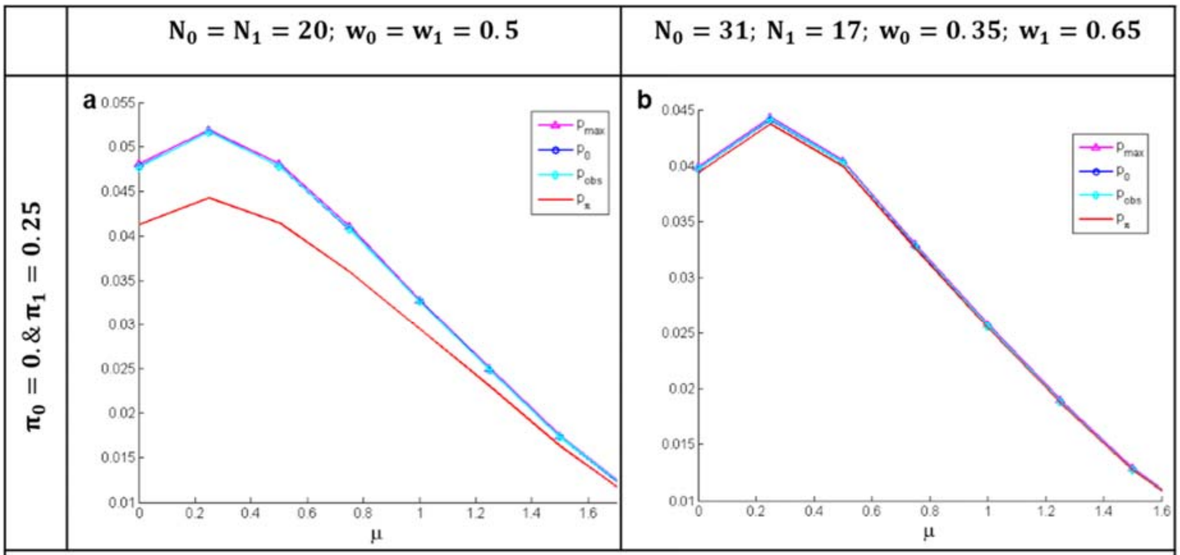

Figure S3 Average p-value ($p \leq 0.1$) for the dissonant case

Figure S3 illustrates the average p-value, given the value is below 10 %, for the three p-value estimates as well as p_π. The slight spike in the graphs illustrates how XERp first identifies the difference between π_0 and π_1, the difference between the shift direction and these proportions result in a slight loss of power which is quickly regained once the shift is more pronounced. The three p-value alternatives perform equally well with p_{obs} displaying slightly more power. The slight advantage of p_{obs} over the other alternatives will become more pronounced for more accurate estimation of π, as is evident from the red lines indicating the somewhat greater power of p_π.

Figures S4 and S5 illustrate the proportion of times the null hypothesis is rejected given significance levels of 5 and 1 %, respectively. To make the graphs more legible they are not displayed for the entire range of shift values (μ). Once no differences between p-value alternatives are apparent and sufficiently

high rejection rates are achieved (i.e. the average p-values have achieved sufficiently low levels) the graphs are no longer displayed.

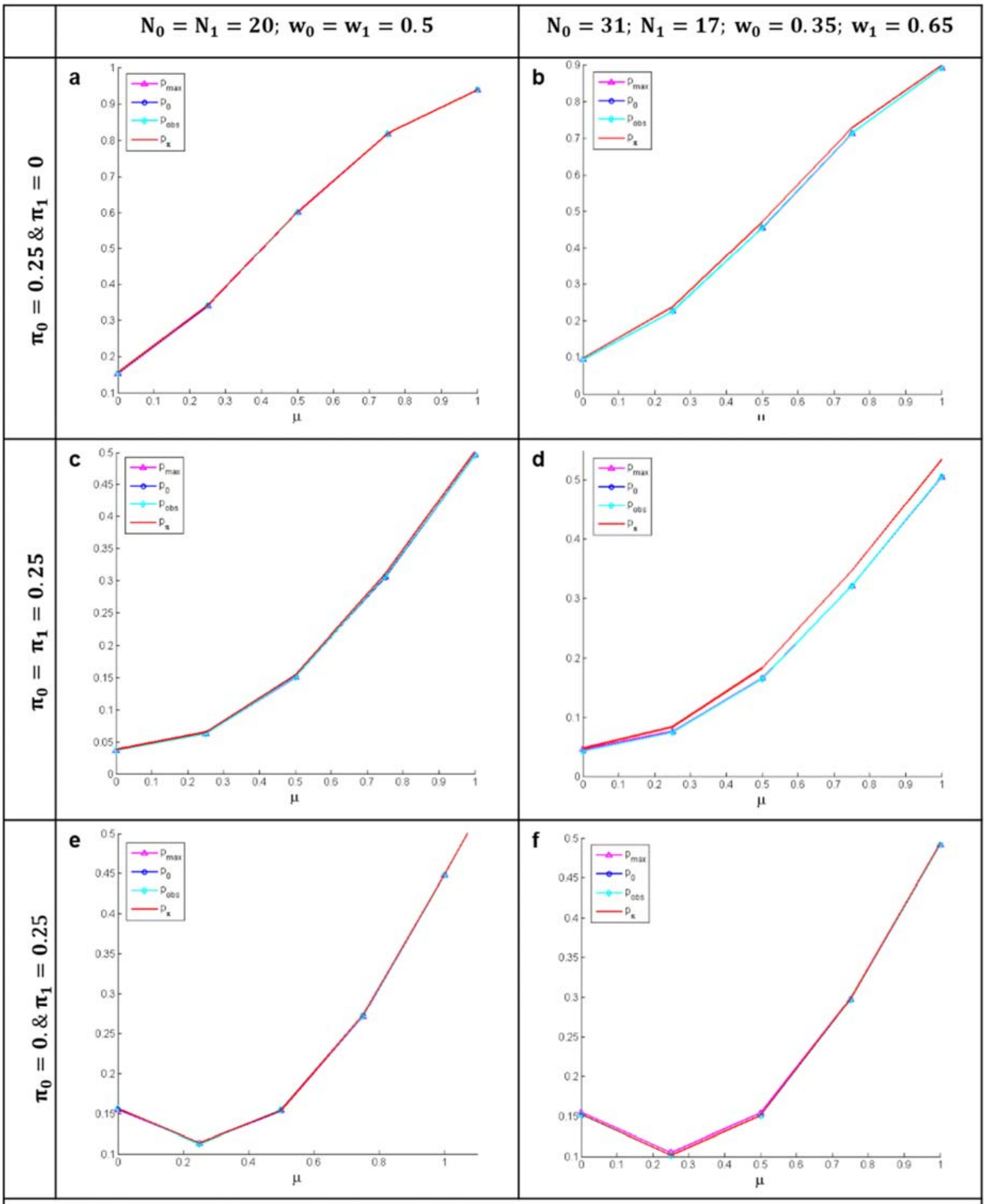

Figure S4 Proportion of null hypothesis rejections given $\alpha = 5\%$

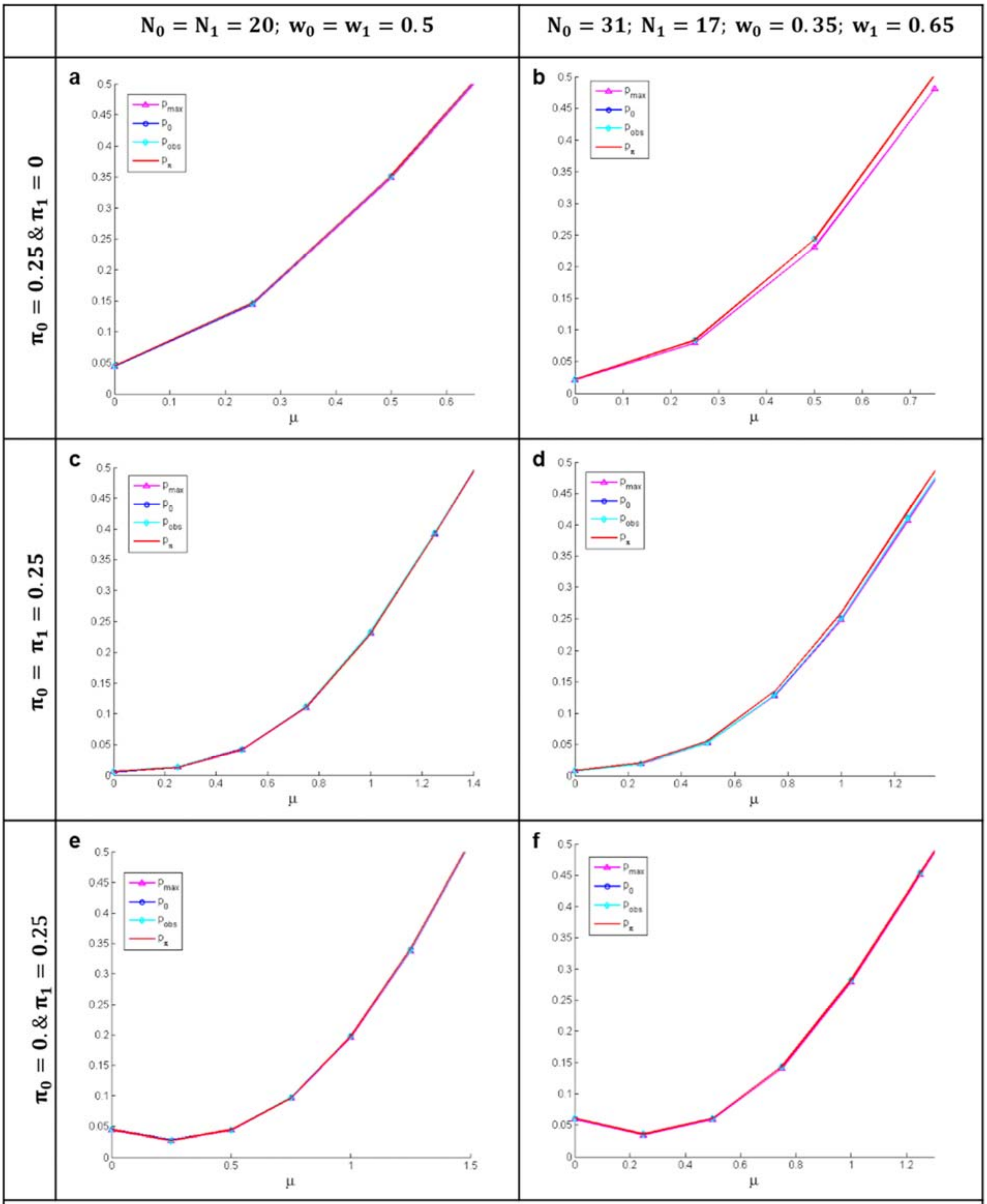

Figure S5 Proportion of null hypothesis rejections given $\alpha = 1\%$

Again the three p-value alternatives perform equally well. Notably, the significance level is retained reasonably well when there is no difference between the groups with $\pi_0 = \pi_1$ and $\mu = 0$ (Figures S4c, S4d, S5c and S5d). XERp again loses some power in the dissonant case, but only until the distributional shift becomes more pronounced.

S7. Comparison to random imputation

This section takes a quick look at a random imputation strategy for replacing zero values and the use of XERp. The simulation made use of three variables selected based on their XERp results as provided in Table S2.

Variable	ER	C	Direction	Observed p-value	Observed π		
					Control	Experimental	All
3,4-dihydroxyphenylacetic acid	0.24	0.99	Up	0.01	29%	18%	25%
2,6-dihydroxybenzoic acid	0.27	0.00	Up	0.05	55%	18%	42%
4-hydroxycyclohexanecarboxylic acid	0.24	0.00	Down	0.01	35%	82%	52%

Table S2. XERp results

From Table S2 we see that the variables are well balanced with regard to the placement and proportion of zero values. The p-values calculated by XERp round up to significance levels of 1 and 5 % meaning that, for illustrative purposes, the selection of the variable is sensitive to small fluctuations in the p-value calculated. Finally, both up- and downward shift scenarios are represented, as well as both threshold scenarios $c = 0$ and $c > 0$.

To perform the simulation, the three variables were passed to the XERp software in two forms: (i) data with zeros (as was done in the main paper and corresponding to the results in Table S2); and (ii) data with zeros replaced by random values from a beta distribution, after which ERp [1] was applied. To average out the impact of random imputation a hundred thousand iterations were carried out. During each iteration random numbers were generated from a beta distribution with location and scale parameters set to half the minimum and the minimum of the non-zero data, respectively, while the shape parameters were both set equal to 2.

The XERp estimated threshold for 3,4-dihydroxyphenylacetic acid is greater than zero, therefore the threshold and error rate are not affected by random imputation of zero values. The same XERp error rate as for the data with zeros of 0.2434 is obtained. The associated p-values did however, differ as different null distributions were referenced, i.e. where there are 25 % zeros $p_{obs} = 0.0123$ (data with zeros) and no zeros $p_{obs} = 0.0126$ (random imputation). It is important to note that in instance where $c > 0$ for the data

with zeros, the error rates will only be equal if the imputed numbers are not allowed to exceed the minimum of the non-zero values. More advanced imputations strategies such as k-nearest-neighbour and random forest may not conform to this rule and will produce different results. Also, in the event that the error rates are the same, the p-value for the data with zeros may not always be smaller as it depends on the proportion of zeros (refer to Figure 2 in the main manuscript).

The results for the second and third variables, 2,6-dihydroxybenzoic acid and 4-hydroxycyclohexanecarboxylic acid, are very similar since both have thresholds $c = 0$ for the data containing zeros (Table S2). In instances where the XERp estimated threshold is equal to zero, ERp combined with imputation by random numbers smaller than the minimum non-zero value, will result in a threshold estimate somewhere between two imputed numbers. In other words, the classification rule depends on some random value between the ranges set by the imputation rule, e.g. zero and the minimum observed value. A threshold that falls in random territory introduces unwanted randomness into the associated error rate and p-value. Most importantly, a threshold in random territory implies that a new subject with a measured value of zero, therefore requiring imputation, would be classified by comparing the random value from imputation to the threshold value, which is also based on random imputations. Classification is thus based on whether one random number is larger or smaller than another random number.

Concluding, imputation with random numbers has no effect on the error rate when the XERp threshold is already above the detection limit, while it can have a dramatic effect when the XERp threshold value is lower than the detection limit. Moreover, random imputation introduces unwanted randomness into the thresholds, error rates and p-values without any obvious gain.

References

[1] Van Reenen M, Reinecke CJ, Westerhuis JA, Venter JH: Variable selection for binary classification using error rate p-values applied to metabolomics data. BMC Bioinformatics. 2016;17(33).

[2] MATLAB and Statistics Toolbox Release 2012b, The MathWorks, Inc., Natick, Massachusetts, United States
